# Shrinkage Cracking Characteristics and Micro-Mechanism of Bentonite and Glass-Fiber-Modified Cement Soil in Dry Environment

**DOI:** 10.3390/ma19081671

**Published:** 2026-04-21

**Authors:** Zili Dai, Xiaowei Lu, Lin Wang, Shifei Yang, Rong Wang

**Affiliations:** 1 Department of Civil Engineering, Shanghai University, 99 Shangda Road, Shanghai 200444, China; zilidai@shu.edu.cn (Z.D.); 17769733317@shu.edu.cn (X.L.); 2 SGIDI Engineering Consulting (Group) Co., Ltd., Shanghai 200093, China; yangshifei@sgidi.com (S.Y.); wangrong@sgidi.com (R.W.); 3 Shanghai Engineering Research Center of Geo-Environment, Shanghai 200093, China; 4 Key Laboratory of Silicate Cultural Heritage Conservation (Shanghai University), Ministry of Education, Shanghai 200444, China

**Keywords:** cement soil, shrinkage cracking, dry environment, bentonite, glass fiber

## Abstract

In order to investigate the effects of bentonite and glass fiber on the macroscopic mechanical properties and microscopic mechanisms of cement soil in dry environments, a series of laboratory tests were conducted in this study, including drying tests under controlled environments (30 °C, 50% humidity), unconfined compressive strength (UCS) tests, digital image processing technology, and scanning electron microscopy (SEM) analyses. The moisture evaporation law, surface crack development process, UCS variation, and microstructure evolution of cement soil with different mix proportions (bentonite content: 0–9%; glass fiber content: 0–0.5%) were systematically analyzed. The results show that bentonite can significantly enhance the water retention capacity of cement soil, reduce the water evaporation rate, and increase the unconfined compressive strength by filling internal pores to densify the microstructure. Glass fibers form a three-dimensional network structure in the matrix, exerting a bridging effect to inhibit crack initiation and propagation, and optimize the mechanical properties. The unconfined compressive strength increases significantly with an increase in bentonite content (3–9%), and the optimal fiber content for strength improvement is determined as 0.3%. The synergistic effect of bentonite and fibers optimizes the interfacial bonding force between fibers and the matrix, which remarkably improves the anti-cracking performance of cement soil. Specifically, when the bentonite content is 6–9% and the fiber content is 0.3–0.5%, the cement soil maintains complete integrity after drying, with no obvious cracks on the surface. SEM analysis reveals that the addition of bentonite and fibers inhibits the expansion and connection of internal voids, avoiding the cycle of “void enlargement–stress concentration–crack propagation”. This study provides a scientific basis for the engineering application of cement soil in a dry environment.

## 1. Introduction

Soil stabilization technology, characterized by distinct advantages such as low cost, low energy consumption, and high strength, is widely applied in diverse engineering scenarios, including road construction, slope reinforcement, and foundation pit backfilling [1,2,3]. By introducing cement or other stabilizers to induce physical and chemical reactions, this technology enhances the engineering properties of soil, effectively improving its utilization value and saving substantial resources for engineering projects [4,5].

However, during the actual service life of engineering structures, cement soil is frequently subjected to adverse climatic conditions such as high temperatures and drought. Under such an environment, cement soil is prone to drying shrinkage cracks due to moisture loss [6,7]. Extensive research has been conducted to investigate the effects of dry environments on cement soil performance. For instance, Tollenaar et al. [8] demonstrated, through a series of experiments, that the water content initiating cracking depends on both the initial water content and drying rate. Wang et al. [9] identified drying shrinkage caused by moisture loss as the primary factor inducing cracking in cement-stabilized subgrade layers, establishing a direct correlation between moisture variations and soil cracking. Qian et al. [1] reported that the unconfined compressive strength of cement soil decreases by over 50% after 10 wet–dry cycles, highlighting the severe degradation of mechanical properties under alternating wet–dry environments. Chompoorat et al. [10] explored the shrinkage and cracking characteristics of soft clay stabilized with ordinary Portland cement (OPC) and fly ash (FA) in deep mixing applications, providing valuable insights for addressing shrinkage cracking issues with different stabilizer combinations. Notably, shrinkage cracking not only results in significant deterioration of cement soil strength and durability but also compromises the stability of engineering structures, posing potential threats to engineering safety [11]. Thus, research into the shrinkage cracking characteristics and micro-mechanism of cement soil in a dry environment bears considerable practical engineering significance.

With the rapid advancement of materials science, modification of cement soil using novel materials (e.g., fibers, resins, polymers) has been proposed to enhance its strength and durability in complex environments [12,13,14]. For example, Dhakal et al. [15] demonstrated that modified cohesive soil with 10% calcium sulfoaluminate cement (CSAC) and 1% fibers maintained high unconfined compressive strength after wet–dry cycles. Wu et al. [16] found a positive correlation between polymer content and the water stability of fiber–polymer-stabilized loess. Liu et al. [17] improved the crack resistance of cement soil by incorporating microbial biopolymers and palm fibers. These studies collectively confirm that adding appropriate additives can significantly enhance the strength and durability of cement soil in complex environments. Nevertheless, there remains a lack of quantitative analysis on the anti-cracking mechanism of modified cement soil [18]. Further research is still required to determine the optimal mix ratio of cement soil and modifiers for adaptation to dry environments.

To address the drying shrinkage cracking issue of cement soil in arid environments, this study proposes a synergistic modification method using bentonite and glass fiber to improve the crack resistance of cement soil. A series of controlled drying tests is carried out under typical dry climatic conditions, and digital image processing technology is adopted to quantitatively characterize the dynamic processes of moisture evaporation, crack initiation, propagation, and morphology evolution. The effects of bentonite and fiber content on anti-cracking performance are systematically analyzed, and the optimal mix proportion for crack-free cement soil after drying is determined. Furthermore, scanning electron microscopy (SEM) is employed to reveal the microscopic mechanism of pore evolution, fiber bridging, and bentonite–fiber synergy in inhibiting shrinkage cracking. This study not only enriches the quantitative evaluation method for the drying cracking of modified cement soil but also provides a theoretical basis and technical support for the engineering application of soil stabilization technology in dry regions.

## 2. Materials and Methods

### 2.1. Experimental Materials

**Figure 1:**
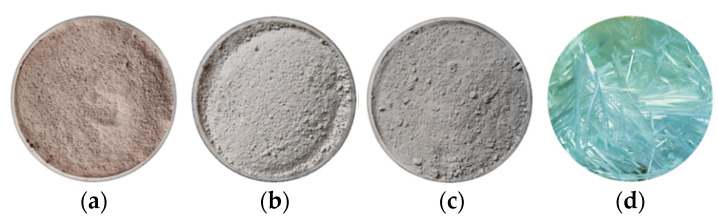
Appearance of experimental materials. (**a**) Excavated soil. (**b**) Bentonite. (**c**) Cement. (**d**) Glass fiber.

The main experimental materials used in this study are shown in Figure 1. Soil was obtained from a construction site in Shanghai, China, and was dried, crushed, and sieved through a 2 mm sieve before tests, as shown in Figure 1a. The fundamental physical properties were tested and are presented in Table 1. The soil had a liquid limit of 46.7% and a plastic limit of 22.9%, classified as a low-liquid-limit clay according to the standard for engineering classification of soil (GB/T 50145-2007) [19]. The mineralogical composition of the soil was measured by X-ray diffraction (XRD), which mainly consisted of quartz (48.8%), muscovite (20.9%), albite (14.4%), kaolinite (8.8%), and chlorite (7.1%). Cement (denoted as C) was adopted as the primary soil stabilizer. The P.O.42.5 ordinary Portland cement (OPC) used in this study was manufactured by Liaoning Daying Cement Group Co., Ltd. (Dengta, China), and its appearance is presented in Figure 1b. Bentonite (denoted as B), a high-purity natural sodium-based modifier, was purchased from Zhejiang Fenghong Clay Chemical Co., Ltd., Huzhou, China; and its morphology is shown in Figure 1c. The chemical compositions of OPC and bentonite are provided by the material manufacturers and listed in Table 2. Glass fiber, another modifier employed to enhance the crack resistance of cement soil, was produced by Jiangxi Yuanyuan New Material Co., Ltd., Ganzhou, China, as illustrated in Figure 1d. Its physical and mechanical properties are summarized in Table 3.

### 2.2. Specimen Preparation

The dosages of stabilizers and modifiers are key factors governing the effectiveness of soil stabilization. In existing studies on cement-stabilized soil, cement contents generally range from 5% to 28% [5]. A cement dosage of approximately 15% has been shown to yield a significant strength improvement while remaining economically viable [20,21]. Previous research has also indicated that a bentonite content exceeding 9% tends to result in poor workability of the soil mixture [22]. Furthermore, preliminary experimental evidence suggests that fiber contents above 0.5% may lead to uneven fiber dispersion and the formation of local weak zones, thereby reducing the strength and stabilization performance of cement soil [23,24,25,26]. Accordingly, fiber mass fractions of 0.1%, 0.3%, and 0.5% were selected in this study to explore their influence on the anti-cracking behavior of cement soil. The mixed proportions of the cement soil specimens are presented in Table 4, noting that all values represent mass fractions relative to the dry weight of soil. The soil matrix is the fundamental component and is not listed separately.

### 2.3. Experimental Device and Method

Based on previous tests, a water–solid ratio of 0.5 was selected. First, soil, cement, fiber, bentonite, and water were weighed according to the mix ratio in Table 4, and the soil, cement, and bentonite were dry-mixed for 30 s. Then, the fiber was added to the dry material in multiple batches and fully stirred to ensure uniform dispersion of the fiber in the dry material. Finally, water was slowly poured in and stirred for 1 min. The mixture was filled into a circular mold with a diameter of 90 mm and a height of 20 mm to ensure good uniformity throughout the macrotest, and then, it was vibrated on a vibration table for 1 min to remove entrapped air bubbles. The surface of the prepared specimens was scraped flat, wrapped with plastic film, and placed in a curing room with a temperature of 20 °C and a humidity of 95% for curing. After curing for 1 day to form, the specimens were demolded and continued to cure for 28 days.

After reaching the specified curing duration, the specimens used for crack observation are taken out of the standard curing room and placed in an environment with a temperature of 30 °C and a humidity of 50% (selected based on Shanghai’s long-term average summer temperature and humidity, the peak period for cement soil cracking in local engineering) for continuous drying. The weight of each sample was measured every two hours—a frequency determined by preliminary tests showing significant mass changes in the specimens every 2 h during the initial 24 h drying stage—to ensure accurate capture of dynamic moisture loss. The drying experiment is terminated when the difference between two adjacent sets of test data is small, i.e., the mass change of the specimen within 4 h is less than 0.1 g.

Unconfined compressive strength (UCS) tests were conducted on specimens before and after drying in accordance with the Chinese GB/T 50123-2019 [27] Standard for Geotechnical Testing Method. A computer-controlled electronic universal testing machine was adopted at a constant displacement rate of 1.0 mm/min. The peak load at failure was recorded automatically, and the UCS value was calculated as the ratio of the maximum compressive load to the cross-sectional area of the specimen.

To analyze the effect of dry environments on the microstructure of cement soil, microstructural scanning was performed on specimens before and after the drying experiment, where the samples for SEM observation were randomly cut from the bulk drying-test specimens (six pieces in total). Since SEM is used to characterize the microscopic morphology and microstructure of cement soil, these small sampled fragments do not require strict macroscopic geometric uniformity, and slight differences in their macroscopic shapes have no impact on the validity and reliability of the microstructural analysis.

For each test in this study, three parallel repetitions were carried out to ensure the reliability and reproducibility of the experimental results. After the completion of each group of tests, the average value of the three parallel test results was taken as the final test data.

In this study, specimens were cured in an SHBY-40B constant temperature and humidity curing box (Wuxi Jianyi Instrument & Machinery Co., Ltd., Wuxi, China), which maintained a constant temperature of (20 ± 2) °C and a relative humidity of ≥95% throughout the curing period, in strict accordance with the relevant specifications for cement-stabilized soil. The unconfined compressive strength (UCS) was determined using a CTM8050 microcomputer-controlled electronic universal testing machine (Shanghai Xieqiang Instrument Co., Ltd., Shanghai, China), with a measurement range of 0–50 kN and an accuracy of ±0.01%. Drying shrinkage and cracking behavior were monitored using a DHG-9070A electric thermostatic drying oven (Yiheng Scientific Instrument Co., Ltd., Shanghai, China), and crack morphology was observed with a VHX-6000 digital microscope (Keyence Corporation, Osaka, Japan). Microstructural characterization was performed using an SU8230 high-resolution cold-field emission scanning electron microscope (SEM) (Hitachi High-Tech Corporation, Tokyo, Japan). X-ray diffraction (XRD) analysis was conducted using an 18 kW D/MAX2500V+/PC X-ray diffractometer (Rigaku Corporation, Tokyo, Japan) at the Analysis and Testing Center of Shanghai University, Shanghai, China.

### 2.4. Crack Image Processing

**Figure 2:**
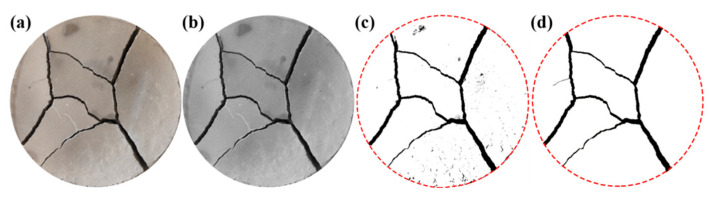
Image processing process: (**a**) original crack image, (**b**) gray scale image, (**c**) image of the crack after binarization, and (**d**) binarization image after removing the spurious points.

During the drying experiment, a camera was positioned above the specimens to document any surface crack developments. To quantitatively analyze the progression of surface cracks during desiccation, the specimen surface image presented in Figure 2a was converted to an 8-bit grayscale, as shown in Figure 2b. Binarization was then performed using Otsu’s algorithm to automatically determine the optimal threshold, which minimizes intra-class variance between the foreground (cracks) and the background (specimen matrix). This yielded a threshold value of 127, where pixel intensities of ≤127 were classified as cracks and those >127 were classified as background (Figure 2c). The threshold was validated by comparing binary outputs with visual inspection of high-contrast regions in the original images to ensure consistent crack segmentation. Subsequent noise elimination was conducted via a two-step process to remove artifacts interfering with surface calculations (Figure 2d): (1) A 3 × 3 median filter was applied to eliminate isolated pixel noise, defined as individual or clusters of ≤3 connected pixels with intensity values inconsistent with adjacent crack features; (2) morphological closing operations using a 2-pixel structuring element were performed to fill minor gaps within crack segments, preserving continuous crack morphology without over-smoothing critical features (e.g., crack tips). These parameters were standardized across all specimens, with their effectiveness verified by comparing quantified metrics with manual measurements on 10 representative sub-regions (mean relative error <5%). The processed image was then analyzed using Image-Pro Plus 9.1, a software chosen for its specialized crack quantification functions, such as accurate measurement of crack length, area ratio, and width parameters [28]. This analysis yielded four key metrics: total crack length (sum of all surface cracks), surface crack ratio (ratio of total crack area to the specimen’s surface area), maximum crack width (the widest dimension of a single crack), and average crack width (calculated as the ratio of total crack area to total crack length). By comparing the pixel width of the specimen image with the specimen diameter, the relevant crack parameters can be calculated. The specific conversion process is outlined as

(1) L=μP,

where *L* is the specimen diameter, *μ* is the conversion factor, and *P* is the pixel width of the specimen image.

## 3. Experimental Results

### 3.1. Water Evaporation Law of Specimens

**Figure 3:**
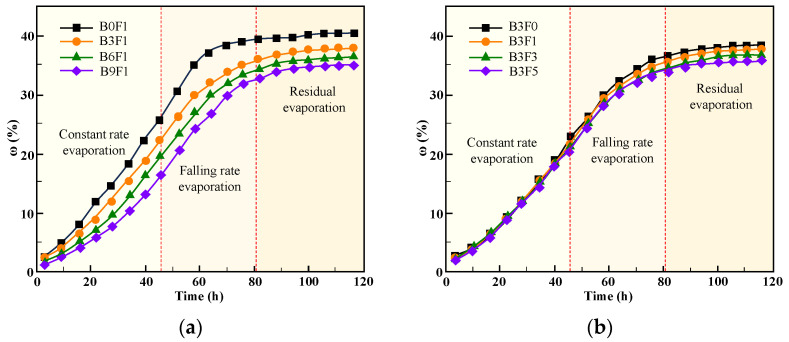
Curves of cumulative evaporation degree over time. (**a**) Cumulative evaporation degree of specimens with different bentonite content. (**b**) Cumulative evaporation degree of specimens with different fiber content.

In a dry environment, the water in specimens absorbs the heat from the atmosphere and evaporates into the environment. Figure 3 presents the curves of evaporation degree versus time for the specimens during the drying test. Throughout the drying process, the cumulative evaporation degree ω of each specimen first increased and then tended to stabilize. The time–evaporation degree curves of the specimens can be mainly divided into three stages, constant-rate evaporation, falling-rate evaporation, and residual evaporation, with their characteristics described as follows: (1) During the constant-rate evaporation stage, the slope of the time–evaporation degree curve remains essentially constant, indicating that the evaporation rate is approximately unchanged with increasing time. (2) In the falling-rate evaporation stage, the slope of the curve gradually decreases, meaning that the evaporation rate decreases as time progresses. (3) During the residual evaporation stage, the slope of the curve gradually decreases from a small value to zero, and the evaporation degree reaches a maximum and then remains constant once the moisture in the specimens is completely evaporated.

By comparing the evaporation degree curves of different specimens, it can be seen that the evaporation rate of specimens decreases significantly with an increase in bentonite content, as illustrated in Figure 3a. The effect of fiber content on the evaporation rate is relatively small. During the constant-rate evaporation stage, the evaporation curves of specimens with different fiber contents almost overlap. In the falling-rate evaporation stage and residual evaporation stage, fibers inhibit the cracking of specimens to a certain extent, thereby reducing the water evaporation rate, as shown in Figure 3b.

The experimental results demonstrate that the addition of bentonite to cemented soil can improve the water retention capacity of specimens in a dry environment. Meanwhile, as shown in Figure 3, for specimens with different fiber contents and a constant bentonite content, increasing the fiber content has no significant influence on the moisture evaporation of specimens.

### 3.2. Fracture Development Law of Specimens

**Figure 4:**
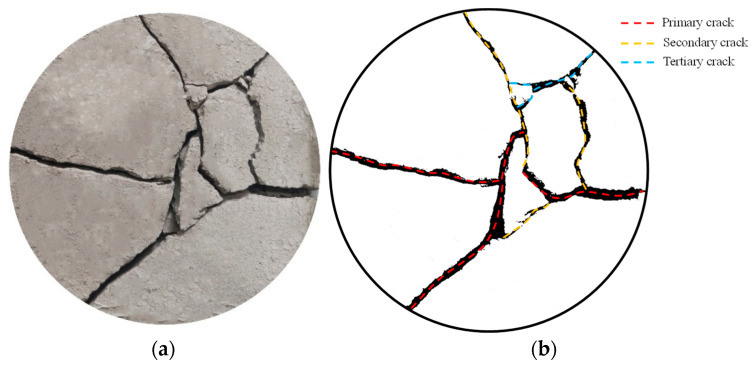
Surface crack morphology of the cement-only admixed specimen (B0F0). (**a**) Original crack image. (**b**) Binarization crack image.

In a dry environment, the moisture in the cement soil evaporates, its volume shrinks, and tensile cracks are prone to occur. Figure 4 illustrates the surface crack morphology of the cement-only admixed specimen (B0F0). The surface cracks of this specimen are complex and interlaced, with a relatively high density of crack intersections. According to the sequence of crack formation, drying shrinkage cracks can be classified into primary, secondary, and tertiary cracks [28]. Their characteristics are as follows: (1) primary cracks extend through the center of the specimen; (2) secondary cracks are perpendicular to the primary cracks; (3) tertiary cracks have a relatively smaller width and intersect with both primary and secondary cracks.

**Figure 5:**
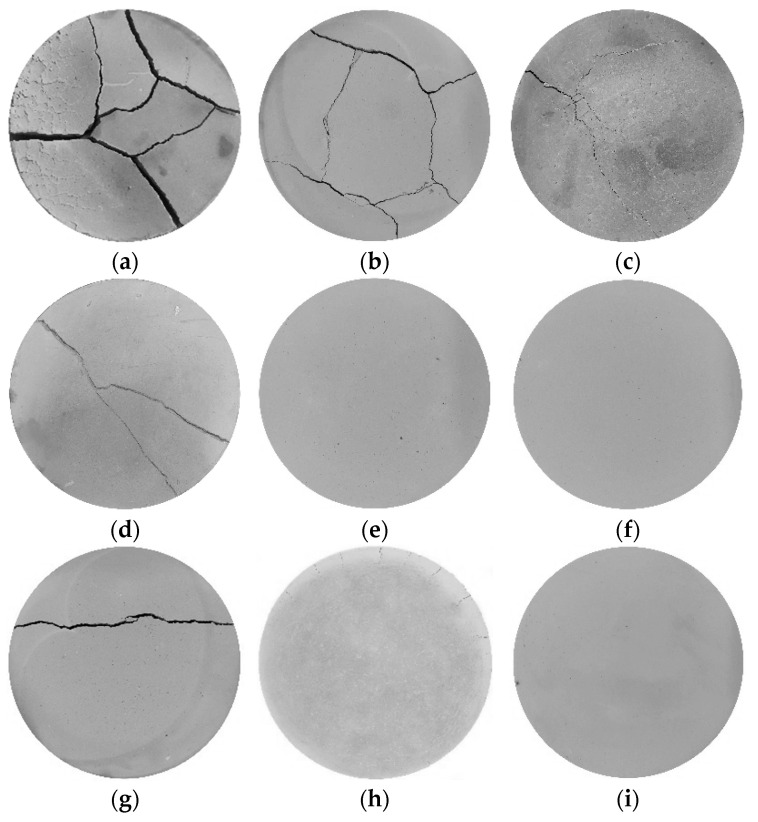
Final crack image of the specimen. (**a**) B3F1. (**b**) B3F3. (**c**) B3F5. (**d**) B6F1. (**e**) B6F3. (**f**) B6F5. (**g**) B9F1. (**h**) B9F3. (**i**) B9F5.

Figure 5 shows the final surface crack development of each specimen. Compared with the cement-only specimens, the number of surface cracks and their intersections in the specimens modified with bentonite and fibers are significantly reduced. When comparing B3F1, B3F3, and B3F5, with an increase in fiber content, the surface crack width of the specimens decreases significantly, accompanied by reductions in the number and length of cracks. In contrast, when comparing B3F1, B6F1, and B9F1, as the bentonite content increases, the number and length of surface cracks decrease remarkably, while the change in crack width is not obvious.

It is also noted that the cement-only specimens in Figure 4 suffered integrity damage after the drying test, with the entire specimens split into soil blocks of varying sizes by the cracks. Similarly, among the specimens modified with bentonite and fibers, when the fiber content is 0.1% (B3F1, B6F1, and B9F1), the specimens also experience integrity failure. However, when the bentonite content is 6% and 9% with fiber contents of 0.3% and 0.5%, the integrity of the specimens remains intact after the drying test.

Based on image processing technology, quantitative analysis was conducted on the crack-related parameters of the specimen surfaces. The final crack ratio (total area of cracks per unit area of the specimen) and crack density (total length of cracks per unit area of the specimen) of each specimen were obtained and compared.

**Figure 6:**
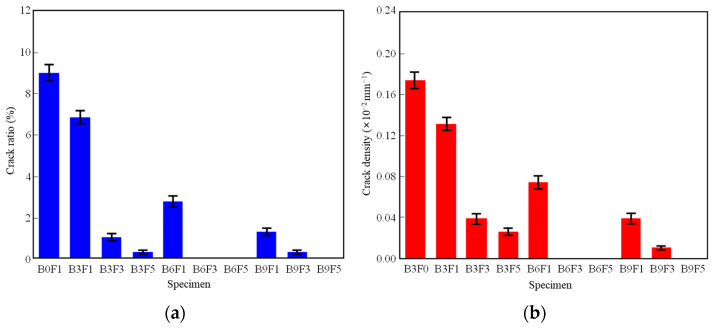
Basic indicators of specimen cracks. (**a**) Crack ratio. (**b**) Crack density.

Figure 6 presents the quantitative indicators of the final cracks for specimens with different mix ratios. Comparing the surface crack ratios of each specimen in Figure 6a, the surface crack ratios of specimens incorporated with bentonite and fibers are significantly reduced compared to the control specimen B0F1. When the fiber content is 0.1%, with an increase in bentonite content, the surface crack ratios of B3F1, B6F1, and B9F1 decrease by approximately 24.0%, 68.5%, and 85.9%, respectively, compared to B0F1. When the bentonite content is 3%, with an increase in fiber content, the surface crack ratios of B3F1, B3F3, and B3F5 decrease by 22.2%, 75.7%, and 86.7%, respectively, compared to B3F0. Meanwhile, after the completion of the drying test, the surface crack ratios of B6F3, B6F5, and B9F5 are all 0, indicating that the cement–bentonite–fiber soil exhibits excellent crack resistance.

Comparing the surface crack densities of each specimen in Figure 6b, when the bentonite content is 3%, the surface crack density after the drying test shows a gradual decrease with an increase in fiber content compared to the cement-only specimen (B3F0), though the crack density of B3F3 remains at a relatively high level. When the bentonite content is 6% and 9%, the crack density of the specimens decreases significantly with the increase in bentonite and fiber contents. Additionally, after the drying test, the surface crack densities of B6F3, B6F5, and B9F5 are all 0, further demonstrating the superior crack resistance of the cement–bentonite–fiber soil.

### 3.3. Unconfined Compressive Strength of Specimens

**Figure 7:**
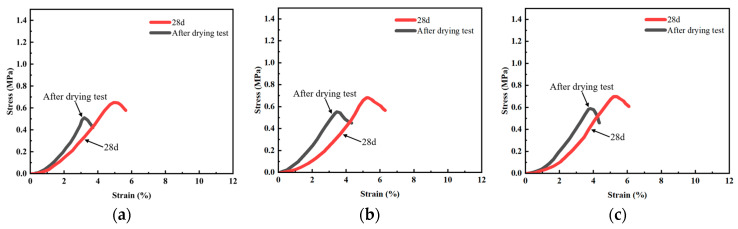

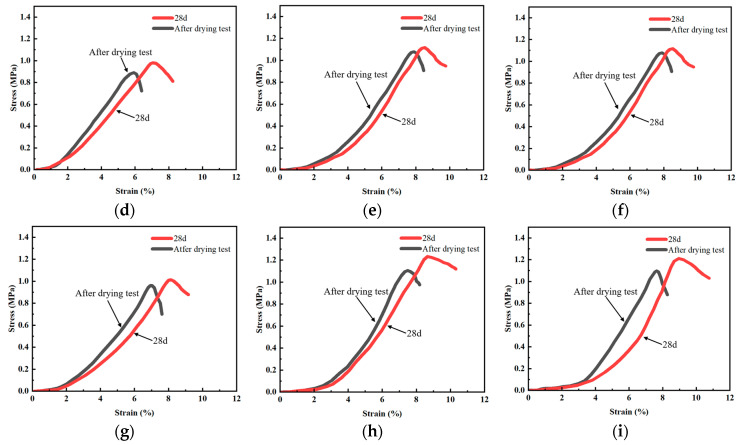
Stress–strain curves of unconfined compressive strength for cement soil specimens before and after drying. (**a**) B3F1. (**b**) B3F3. (**c**) B3F5. (**d**) B6F1. (**e**) B6F3. (**f**) B6F5. (**g**) B9F1. (**h**) B9F3. (**i**) B9F5.

Figure 7 illustrates the stress–strain curves derived from unconfined compressive strength (UCS) tests of various cement soil specimens. It can be seen that after the drying test, the peak stress of the stabilized soil specimens drops considerably, representing obvious strength degradation. Meanwhile, the strain corresponding to the peak stress decreases markedly, which suggests an increased brittleness of the specimens.

#### 3.3.1. Effect of Bentonite Content

The experimental results indicate that bentonite content poses a dominant positive effect on the UCS of the modified cement soil. As the bentonite content rises from 3% to 9% (i.e., from B3 to B9), the peak stress increases continuously and significantly in all fiber-doped groups. Such improvement is mainly attributed to the strong water retention and pore-filling effects of bentonite, which densify the soil matrix, optimize the internal pore structure, and enhance the interfacial bonding between cement hydration products and soil particles. It is worth noting that the growth rate of strength slows down obviously when bentonite exceeds 6%, showing a marginal gain effect. This implies that 6% bentonite can provide sufficient structural reinforcement, while an overlarge dosage (e.g., 9%) may cause particle agglomeration and introduce micro-defects, thus restricting further strength improvement.

#### 3.3.2. Effect of Fiber Content

Fiber contents exert a moderate but crucial influence on the UCS, with an optimal dosage determined in this study. At low bentonite contents (B3), fibers present no distinct strength enhancement, indicating that the matrix needs a certain degree of densification by bentonite to exert the fiber bridging effect effectively. On the contrary, at higher bentonite contents (B6 and B9), the UCS increases sharply as fiber contents rise from 0.1% to 0.3%, with a maximum strength improvement of about 22%. A further increase to 0.5% leads to a strength plateau or a slight decline. This phenomenon demonstrates that a proper fiber dosage can form a stable three-dimensional network structure, which restrains crack initiation and propagation and improves the load-bearing capacity. Nevertheless, excessive fiber tends to agglomerate and disturb uniform mixing, forming weak interfaces and weakening the reinforcing effect. Accordingly, the optimal fiber content for the tested cement soil is recommended as 0.3%.

## 4. Microscopic Reinforcement Mechanism of Cement Soil

In a dry environment, soil moisture loss induces changes in its microstructure. The incorporation of bentonite is capable of slowing down the water evaporation rate, while the bonding between fibers and curing agent can effectively enhance the anti-cracking performance of cement soil. In this study, scanning electron microscopy (SEM) tests were performed to explore the void variations in cement soil in arid environments and the improvement mechanism of fibers and bentonite with respect to the shrinkage cracking of cement soil from a microscopic viewpoint.

### 4.1. Void Variations in Cement Soil in Arid Environments

**Figure 8:**
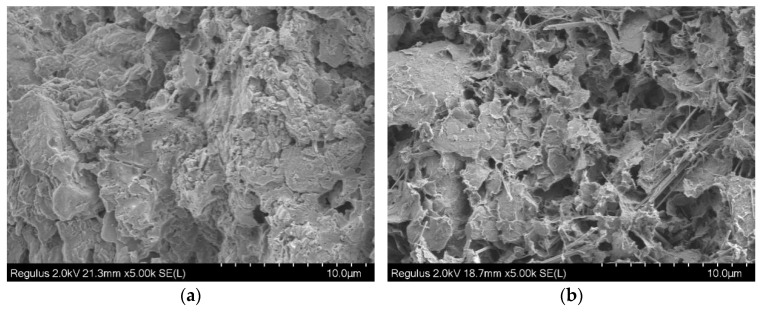
SEM images of specimens before and after drying and dehydration. (**a**) After 28-day curing. (**b**) After drying and water loss.

Figure 8 shows the SEM images of cement soil before and after drying and dehydration. After 28-day curing (Figure 8a), the cement soil exhibits a dense microstructure, where soil particles are tightly bonded with cement hydration gels. The voids are mainly small, dispersed, closed pores, with low porosity and small pore volume. The overall structure has good continuity, and the bonding between particles is strong. After drying treatment, the microstructure changes significantly: (1) The porosity and pore volume increase remarkably, with the original tiny pores connecting and expanding to form a large number of irregular open pores. (2) The structure transforms from a dense state to a loose and porous state, the connections between particles are weakened, and obvious cracks and particle separation appear in some areas.

The SEM images indicate that after drying and dehydration, the porosity and pore volume of the cement soil increase significantly, which is specifically reflected in the transformation of the microstructure from a dense structure (Figure 8a) to a loose and porous structure (Figure 8b). In a dry environment, the evaporation of internal moisture in the cement soil causes the bound water film on the surface of soil particles to thin, generating inward shrinkage forces between particles. In the initial dense structure, small and dispersed pores can transfer stress through the bonding force between particles and gels; however, as moisture continues to be lost, the voids continuously expand and connect to form a loose and porous structure, reducing the effective contact area between particles. The bonding force is insufficient for balancing the shrinkage force, leading to local stress concentration. Meanwhile, the drying shrinkage of cement hydration gels themselves further exacerbates void expansion. When the local stress exceeds the tensile strength of the material, microcracks initiate in the weak zones with concentrated pores and propagate and connect along the loose and porous weak areas. The enlarged voids also provide more channels for moisture evaporation, accelerating water loss and stress concentration, thus forming a cycle of “void enlargement–stress concentration–crack propagation–further void enlargement”, which eventually develops into macroscopically visible shrinkage cracks.

### 4.2. Improvement Mechanism of Bentonite on Cement Soil

**Figure 9:**
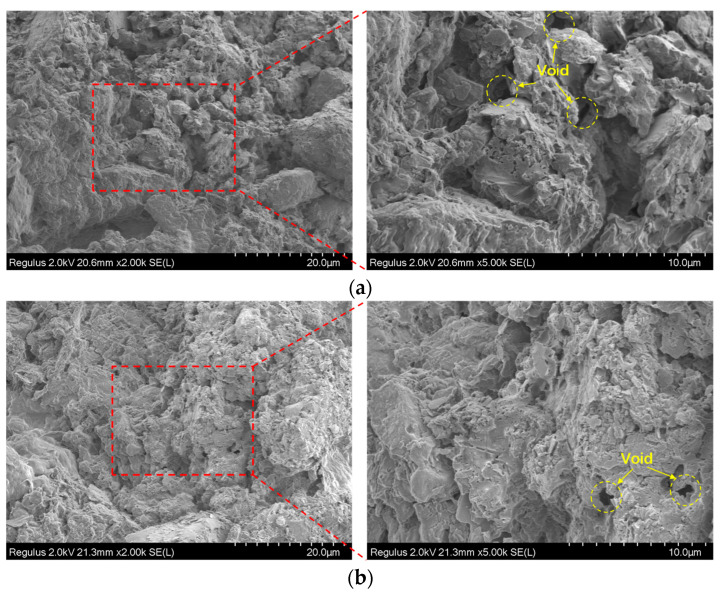

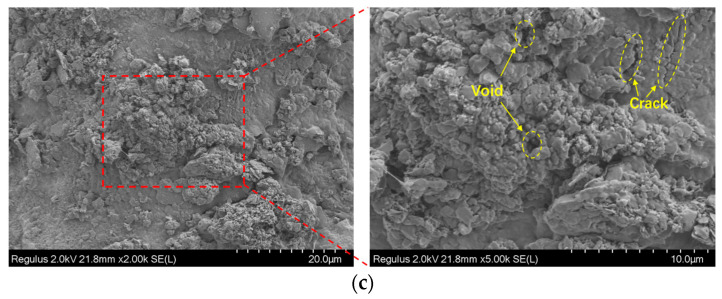
Influence of bentonite content on microstructure: (**a**) 3% bentonite content; (**b**) 6% bentonite content; (**c**) 9% bentonite content.

The effect of varying bentonite contents on the microstructure of cement soil is illustrated in Figure 9. Studies have shown that the gel substances produced by cement hydration mainly serve to cement soil particles in specimens. However, their efficiency in filling pores is relatively low in loose and porous soft soil. Bentonite is primarily composed of montmorillonite, and it mainly functions as a pore-filling agent in cement soil. It can be observed that with the gradual increase in bentonite content, the microstructure of cement soil becomes denser, and its overall structural integrity is significantly enhanced. Therefore, incorporating a certain proportion of expansive materials into cement soil to fill the pores between particles can more effectively improve the strength of cement soil.

However, the bentonite content in the specimens should not be excessively high. Bentonite is characterized by swelling when wet and shrinking when dry, and its volume fluctuates drastically with changes in moisture content. When the bentonite content in the specimens is excessively high, excessive expansion occurs after mixing and casting. Moreover, the increased strain caused by moisture loss during drying elevates the stress induced by shrinkage, which exerts an adverse effect on the durability of the specimens. As can be seen from Figure 9c, an excessive amount of bentonite leads to local agglomeration and excessive expansion, which generates microcracks in the cement soil, loosens its microstructure, further impairs the structural integrity of the material, and thus compromises the durability of the specimens.

### 4.3. Improvement Mechanism of Fibers on Cement Soil

**Figure 10:**
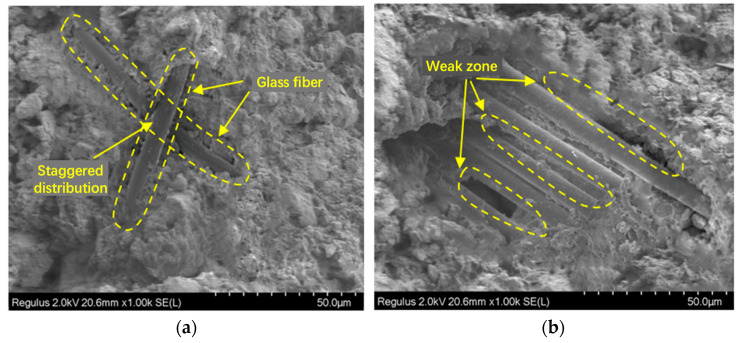
Influence of fibers on microstructure. (**a**) Fiber bridging. (**b**) Weak zone.

Figure 10 shows the scanning electron microscopy (SEM) images of the specimens with fiber addition. As observed from Figure 10a, the glass fibers exhibit a three-dimensional staggered distribution in the specimens, and the surface of the glass fibers is tightly bonded to the cement soil matrix. Owing to the excellent heat resistance and chemical stability of glass fibers, when the specimens crack due to shrinkage caused by water loss in a dry environment or under load, the glass fibers can exert a bridging effect, thereby enhancing the strength and crack resistance of the specimens.

The reinforcement improvement effect of fibers in the cement soil can be explained by the bonding strength, interlocking force, and static friction between the fiber surface and the cement soil matrix [29]. These three interactions enable the fibers to utilize their high tensile strength to prevent or delay the formation and propagation of tensile cracks when the specimens are under load. However, excessive fiber agglomeration will reduce the effective contact area between the fibers and the particles, further leading to the formation of weak zones, as illustrated in Figure 10b. Previous studies have also reported that excessive fiber content induces the emergence of weak zones, exerting an adverse effect on the performance of specimens [30].

**Figure 11:**
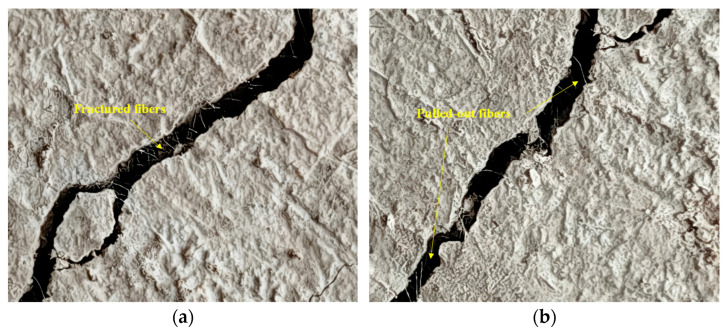
Fiber function failure in cement soil. (**a**) Fiber fracture. (**b**) Fiber pull-out.

Owing to the high tensile strength and good durability of glass fibers, the fibers bear the tensile stress induced by cracking when the specimens crack under load or due to drying shrinkage. When the external load or drying shrinkage stress exceeds the tensile strength of the fibers or when the interfacial force between the fibers and the matrix is insufficient for resisting these external forces, the function of the fibers will fail. Such failure is mainly manifested in two forms: one is that the tensile stress of cracking exceeds the tensile strength of the fibers, leading to fiber fracture; the other is that relative displacement occurs between the fibers and the matrix, the bonding force between the fibers and the matrix fails, the static friction is converted into dynamic friction, and finally one end of the fibers is pulled out from the matrix, as shown in Figure 11.

### 4.4. Synergistic Modification Mechanism of Bentonite-Fiber

In the original soil samples, the particles exhibit a packed arrangement and are primarily connected via contact-type bonds, with relatively large pores existing between them. Cement generates gels through hydration reactions, which coat the surface of soil particles, cement them together, and thus enhance the inter-particle bonding. The gels produced by cement hydration cover the surface of soil particles and serve to wrap and cement the particles, thereby improving the bonding strength between them. However, in loose and porous soft soils, the effect of cement is mainly concentrated on wrapping and cementing soil particles, with a relatively low efficiency in pore filling [31].

In cement soil, fibers are randomly distributed to form a three-dimensional network structure, which helps enhance the tensile strength. Studies have shown that the interfacial force between fibers and the matrix is affected by multiple factors, such as effective contact area, properties of cementitious materials, and moisture content [32]. Especially when the matrix has a high porosity, the friction and bonding force between fibers and the matrix will be weakened, which results in relative displacement between the matrix and fibers when the specimens are subjected to tensile loads. During the cracking process, fibers are likely to be pulled out rather than fractured [33].

Bentonite can effectively fill the pores. It can be clearly observed through the analysis of microscopic images that after the incorporation of bentonite, the microstructure of the specimens becomes much denser, consistent with the findings of Guarena et al. [34]. The pore-filling effect of bentonite enhances the interfacial force between fibers and the matrix. The synergistic effect between bentonite and fibers significantly improves the mechanical properties and durability of the specimens.

### 4.5. Advantages and Limitations of SEM

In the present microstructural investigation, scanning electron microscopy (SEM) delivers unique and irreplaceable advantages for unraveling the microscopic mechanism underlying the drying shrinkage cracking of cement soil. Operating at high magnification, SEM enables clear visualization of soil particles, cement hydration products, bentonite agglomerates, and glass fibers at submicron to micron scales, supporting direct identification of pore dimensions, geometry, connectivity, and spatial distribution. It distinctly captures the pore-filling effect of bentonite, the three-dimensional network distribution and bridging action of glass fibers, and the interfacial bonding condition between fibers and the matrix, supplying direct morphological evidence for clarifying the synergistic modification mechanism. Moreover, SEM supports reliable comparative analysis of microstructural variations between specimens before and after drying, clearly revealing the transition from a compact structure to a loose and porous state and the initiation of microcracks, which provides strong validation for macroscopic mechanical and cracking test data.

Nevertheless, SEM presents inherent scale limitations in crack analysis. The magnification employed in this study exceeded 1000×, which allows clear identification of micropores, fiber–matrix interfacial bonding, and microcracks, but it is unable to characterize the full morphology, connectivity, and spatial distribution of macroscopic cracks. Meanwhile, SEM only provides static snapshots of the microstructure before and after drying, rather than continuously tracking the dynamic evolution of void expansion, interfacial debonding, and microcrack propagation during the drying process. Furthermore, SEM images only reflect local micro-regions, and sample preparation procedures such as cutting, drying, and gold spraying may introduce artificial microcracks and structural disturbances, which, to some extent, weaken the representativeness and authenticity of the microscopic observations.

## 5. Conclusions

This study conducted a series of indoor evaporation experiments to investigate the shrinkage cracking process of cement soil in dry environments, and combined with scanning electron microscopy (SEM) tests, we analyzed the microstructure changes caused by soil moisture loss, as well as the improvement mechanism of bentonite and fibers on this issue. Based on the above analysis, the main conclusions are drawn as follows:

(1) In the drying process, the cumulative evaporation degree of cement soil first increases rapidly and then tends to stabilize, undergoing three stages: constant-rate evaporation, falling-rate evaporation, and residual evaporation. Bentonite can significantly improve the water retention capacity of cement soil—with an increase in bentonite content, the evaporation rate in the constant and falling-rate stages decreases, and the final cumulative evaporation degree is reduced. However, fiber content has no significant effect on the moisture evaporation law of cement soil under the test conditions.
(2) Digital image processing technology was adopted to quantitatively characterize the surface crack parameters (crack ratio, crack density, crack length, and width) of cement soil, which accurately revealed the fracture development law of cement soil under a dry environment. The cement-only cement soil (B0F0) suffers severe integrity damage after drying, with complex and interlaced surface cracks. The incorporation of bentonite and fibers significantly reduces the number, length, and width of cracks. When the fiber content is 0.1%, increasing bentonite content from 0% to 9% reduces the surface crack ratio by 85.9% (compared to B0F1). When the bentonite content is 3%, increasing fiber content from 0% to 0.5% reduces the surface crack ratio by 86.7% (compared to B3F0). The quantitative results of image analyses show that the optimal mix proportion is 6–9% bentonite and 0.3–0.5% fiber, where the cement soil maintains complete integrity without visible cracks after drying.
(3) Unconfined compressive strength tests indicate that bentonite content poses a dominant positive effect on the strength of modified cement soil. As bentonite increases from 3% to 9%, the peak stress rises continuously, while the strength growth rate slows down when bentonite exceeds 6%. Glass fiber exhibits an optimal dosage of 0.3%: At higher bentonite contents (6% and 9%), fibers significantly improve the compressive strength; excessive fiber (0.5%) causes agglomeration and weak interfaces, resulting in a strength plateau or slight decrease. After drying, all specimens show an obvious decrease in peak stress and corresponding strain, indicating strength degradation and increased brittleness.
(4) Bentonite, as a pore-filling agent, densifies the microstructure of cement soil and enhances interfacial bonding between fibers and the matrix, but excessive content (>9%) may cause agglomeration and microcracks. Glass fibers form a three-dimensional network, exerting a bridging effect to resist tensile stress; their failure modes include fracture and pull-out. The synergistic effect of bentonite and fibers inhibits void expansion and connection, reduces stress concentration, and blocks the formation and propagation of macrocracks.
(5) Combined with quantitative image analysis and SEM microscopic testing, the bentonite–fiber modification method can effectively mitigate the shrinkage cracking phenomenon of cement soil in dry environments, improving its durability and engineering applicability. This study provides a practical technical pathway for the resourceful reuse of soft soil in engineering projects in arid regions.

## Figures and Tables

**Table 1:** Physical characteristics of engineering excavated soil.

Water Content (%)	Liquid Limit (%)	Plastic Limit (%)	Plastic Index (/)	Density (g/cm^3^)
23.5	46.7	22.9	23.8	1.81

**Table 2:** Chemical compositions of ordinary Portland cement (OPC) and bentonite (mass fraction, %).

Component	CaO	SiO_2_	Al_2_O_3_	Fe_2_O_3_	H_2_O^+^	H_2_O^−^	FeO	MgO	K_2_O	Na_2_O	SO_3_	f-CaO
OPC	59.9	23.96	6.34	3.46	-	-	-	0.97	-	-	2.14	1.6
Bentonite	1.32	54.1	18.4	1.6	4.58	12.61	0.34	2.57	0.45	1.86	-	-

**Table 3:** Physical and mechanical properties of glass fiber.

Length/mm	Tensile Strength/MPa	Elastic Modulus/MPa	Elongation at Break/%	Melting Point/°C
9	427	4100	45	175

**Table 4:** Mix proportions of cement soil specimens (mass fractions relative to dry soil).

Sample	Dosage/%		
	Cement	Bentonite	Glass Fiber
B0F0	15	0	0.0
B0F1	15	0	0.1
B3F0	15	3	0.0
B3F1	15	3	0.1
B3F3	15	3	0.3
B3F5	15	3	0.5
B6F1	15	6	0.1
B6F3	15	6	0.3
B6F5	15	6	0.5
B9F1	15	9	0.1
B9F3	15	9	0.3
B9F5	15	9	0.5

## Data Availability

The original contributions presented in this study are included in the article. Further inquiries can be directed to the corresponding author.
